# Childhood Asthma Management Pre- and Post-Incident Asthma Hospitalization

**DOI:** 10.1371/journal.pone.0076439

**Published:** 2013-10-18

**Authors:** Marina Bianchi, Antonio Clavenna, Marco Sequi, Angela Bortolotti, Ida Fortino, Luca Merlino, Maurizio Bonati

**Affiliations:** 1 IRCCS - Istituto di Ricerche Farmacologiche Mario Negri, Department of Public Health Laboratory for Mother and Child Health, Milan, Italy; 2 Regional Health Ministry, Lombardy Region, Milan, Italy; Nottingham University, United Kingdom

## Abstract

Many hospitalizations for asthma could potentially be avoided with appropriate management. The aim of this study was to analyze data on disease management of a paediatric population with a hospitalization for asthma. The study population comprised 6–17 year old subjects belonging to three local health units of the Lombardy Region, northern Italy. Regional administrative databases were used to collect data on: the number of children with an incident hospitalization for asthma during the 2004–2006 period, anti-asthma therapy, specialist visit referrals, and claims for spirometry, released in the 12 months before and after hospitalization. Each patient’s asthma management profile was compared with GINA guideline recommendations. Among the 183 hospitalized subjects, 101 (55%) received therapy before hospitalization and 82 (45%) did not. 10% did not receive any therapy either before or after hospital admission and in 13% the therapy was discontinued afterward. Based on GINA guidelines, asthma management adhered to recommendations only for 55% of subjects. Results may suggest that for half of hospitalized subjects, inaccurate diagnosis, under-treatment/scarce compliance with asthma guidelines by physicians, and/or scarce compliance to therapy by patients/their parents occurred. In all these cases, hospitalization would be a proxy indicator of preventable poor control of disease, rather than a proxy indicator of severity.

## Introduction

Asthma belongs to the group of those so-called ‘ambulatory care sensitive conditions’ for which hospital admission [1], [2] and readmission [3], [4] could be prevented by interventions in primary care. According to the Global Initiative for Asthma (GINA) guidelines on asthma management and prevention [5], well-treated/managed patients are expected to not only have minimal chronic symptoms, but also no emergency visits and/or hospitalizations. Regular doctor visits for the assessment of asthma control, which includes the evaluation of the likelihood of asthma exacerbations and educational reinforcement on the importance of compliance with maintenance therapy, are recommended. Although hospital admissions for asthma are not necessarily a marker of poor performing, when asthma requires hospitalization it should be necessary to distinguish between lack of disease control due to severe/difficult-to-treat cases and non adequately treated/managed cases.

While in adults symptoms worsen prior the onset of acute exacerbations [6], children who are at risk of exacerbations from asthma may not experience aggravation of symptoms before the onset of the severe exacerbation [7]. In children, the strongest predictors of risk of exacerbations are a history of previous exacerbation [8], emergency department visit and/or hospitalization, a persistent airflow obstruction measured by spirometry [9].

The aims of this study were to select a population of children who underwent an incident hospitalization for asthma, analyze the extent of compliance to GINA guidelines of its anti-asthma drug prescription profiles and disease management one year prior to, and one year after, the hospital admission. The novelty of this study is the use of administrative database to obtain a proxy of asthma hospitalization due to non adequately treated/managed cases.

## Methods

### Data Source

We interrogated health administrative databases for 742.368 children between 6–17 years of age residents of three representative local health units in the Lombardy region as described previously [10]–[12]. The use of administrative database has been previously validated [13]. Patient records were included in the study for children who had an incident hospital discharge diagnosis of asthma (code 493 of the ICD-9-CM) between January 2004 and December 2006, and having at least one of the following: anti-asthma drug prescription, spirometry claim or specialist visit referral released 12 months before and/or after incident hospitalization. Incident hospitalization was defined as a hospitalization for asthma occurring after a period of 24 months free of hospitalization for asthma. Prescriptions of anti-asthma drugs belonging to the R03 main therapeutic group of the Anatomical Therapeutic Chemical classification system (only age-appropriate formulations were considered: metered dose inhaler or dry powder inhaler, and not nebulized formulations), referrals for specialist visits and claims for spirometry testing performed in hospital outpatient ambulatories 12 months pre- and 12 months post-incident hospitalization were retrieved. A specialist visit was defined as a visit to a pneumologist or allergologist. Since spirometry testing may be performed during a specialist visit, the number of subjects receiving one or the other referral was taken into consideration (‘spirometry and/or specialist visit’). Readmission was defined as a hospitalization for asthma occurring within a period of 12 months after the incident hospitalization. ‘Add on’ therapy was defined as an increase in number of controller drugs from among: long acting ß–agonists (LABA), leukotriene receptor antagonists (LTRA), cromolyn sodium, nedocromil, and methylxanthines. ‘Appropriateness’ was defined as maintenance or step up of therapy after hospitalization and performance of spirometry and/or visit prior to, and after, hospitalization. Introduction of therapy and performance of spirometry and/or visit only after hospitalization was defined as ‘potential appropriateness’ because asthma diagnosis could have been made at the time of hospitalization.

### Statistical Analyses

Statistical analyses were performed using SAS software, version 9.1 (SAS Inc., Cary, NC, USA).

A chi-square test was performed to compare the primary care modifications (therapy, specialist visit referrals and spirometry claims) after versus prior to the incident hospitalization.

### Ethics Statement

This is a population-based observational analysis in which data sources were health administrative databases of the Lombardy Region, and were managed and analysed using an anonymous patient code. Prof. Silvio Garattini, in charge of the Institutional Review Board, issued a formal written waiver for the need of ethics approval.

## Results

During the 2004–2006 observational period, 183 youths (110 boys and 73 girls) of the 742,368 residents met the inclusion criteria. The median age was 10 (IQR = 8–12 for boys, 9–13 for girls), the median length of hospitalization was 4 days (IQR = 3–5), and 87% of the subjects were hospitalized in the pediatric ward. A summary of subjects receiving anti-asthma therapy, spirometry claims, and specialist visit referrals is reported in Table 1.

**Table 1:** Drug therapy and management prior to, and after, hospitalization (n = 183).

	Pre		Post	
	N *(%)*		N *(%)*	
**Drug therapy**		−82 *(45)*	−18 *(10)*	Never received
			+64 *(35)*	Introduced
		+101 *(55)*	−24 *(13)*	Discontinued
			+77 *(42)*	Maintained*
**Spirometry**		−143 *(78)*	−102 *(56)*	Never tested
			+41 *(22)*	Post only
		+40 *(22)*	−9 *(5)*	Pre only
			+31 *(17)*	Pre and post
**Specialist visit**		−157 *(86)*	−125 *(68)*	Never visited
			+32 *(18)*	Post only
		+26 *(14)*	−12 *(7)*	Pre only
			+14 *(7)*	Pre and post
**Spirometry and/or Specialist visit**		−127 *(69)*	−75 *(41)*	Never tested/visited
			+52 *(28)*	Post only
		+56 *(31)*	−13 *(7)*	Pre only
			+43 *(24)*	Pre and post

Pre = pre-hospitalization; Post = post-hospitalization.

* therapy maintained without ‘add on’ (59), and with ‘add on’ (18).

### Anti-asthma Therapy

During the 12 months before the hospitalization, 55% of the children received anti-asthma therapy and 45% did not.

During the 12 months after the hospitalization, among the 101 subjects who had received therapy before hospitalization, 77 continued to receive drug therapy. Of these 77 children, 39 continued to receive the same treatment, 20 underwent therapy modifications without ‘add on’ therapy, and 18 received an ‘add on’ controller drug, mainly LABA and LTRA (Table 2).

**Table 2:** Modification of anti-asthma therapy after incident hospitalization for asthma (subjects in therapy before hospitalization n = 77).

	Pre	Post	p-value
	N *(%)*	N *(%)*	
**with SABA**	64 *(83.0)*	65 *(84.0)*	
**SABA only**	7 *(9.1)*	0 *(−)*	**0.0234**
**SABA+ ICS**	15 *(19.5)*	8 *(10.4)*	
**SABA+ ICS+add on:**			
**LABA**	25 *(32.5)*	27 *(35.1)*	
**LABA+LTRA**	8 *(10.4)*	22 *(28.6)*	**0.0082**
**other**	9 *(11.7)*	8 *(10.4)*	
**without SABA**	13 *(17.0)*	12 *(16.0)*	
**ICS only**	4 *(5.2)*	1 *(1.3)*	
**ICS+other**	9 *(11.7)*	11 *(14.3)*	

Pre = pre-hospitalization; Post = post-hospitalization.

Other = LTRA, cromolyn sodium, nedocromil, methylxanthines.

Among the 82 subjects not treated before hospitalization, in 64 a therapy had been introduced during the 12 months after hospitalization. The first therapy prescribed was: short-acting ß-agonists (SABA), mainly salbutamol (38%); LABA+ICS, mainly salmeterol+fluticasone (32%); ICS, fluticasone or beclometasone (21%); ICS+SABA, beclomethasone+salbutamol (7%); LABA alone, salmeterol (2%).

### Spirometry Testing and Specialist Visits

During the 12 months before the hospitalization, 69% of hospitalized children did not receive spirometry claims and/or visit referrals (Table 1). During the 12 months after the hospitalization, half of subjects did not receive spirometry claims and/or visit (referrals (Table 1). After hospitalization, a total of 72 children (39%) received a spirometry claim, 48 of whom were boys and 24 girls, M/F = 2.0, with a median age of 11.6 (IQR 10–13). A total of 46 subjects (25%) received a specialist visit referral, 27 of whom were boys and 19 girls, M/F = 1.4, with a median age of 11.6 (IQR 9–14). Lastly, 95 (52%) children received a spirometry and/or a specialist visit after hospitalization, 61 of whom were boys and 34 girls, M/F = 1.8, with a median age of 10.0 (IQR 8–13).

### Appropriateness

In order to identify subjects receiving a disease management as recommended by the GINA guidelines, in Table 3 the post-hospitalization drug therapy modification of subjects receiving or not receiving spirometry and/or specialist visits is reported. Group 1 (44% of those hospitalized) comprised the subjects whose management was potentially non-adherent to guideline recommendations, i.e. subjects who: never received drug therapy, albeit they received spirometry and/or a specialist visit (9.8%), received drug therapy only after hospitalization, albeit spirometry or pre-hospitalization visit could have led to a diagnosis (4.4%), subjects whose therapy had been discontinued (13.1%), or those continuing therapy, but without spirometry or a visit (17.5%). Group 2 (31% of those hospitalized) comprised the subjects potentially adherent to guideline recommendations, who received drug therapy only after hospitalization and who had not been tested by spirometry, or who had been tested by spirometry after hospitalization. Group 3 (24% of those hospitalized) comprised the subjects adherent to guideline recommendations. These received drug therapy prior to, and after, hospitalization and spirometry/specialist visits prior to, and after, or only after, hospitalization.

**Table 3:** Proxy analysis of adherence to GINA guidelines.

		Drug therapy			
		N *(%)*			
		Never received	Introduced	Discontinued	Maintained
	Never received	**–**	**35 ** ***(19.1)*** §	**11 ** ***(6.0)*** **°**	**29 ** ***(15.8)*** **°**
**Spirometry and/or Specialist visit**	Received Pre and Post	**8 ** ***(4.4)*** **°**	**4 ** ***(2.21)*** **°**	**3 ** ***(1.6)*** **°**	**29 ** ***(15.8)*** *
	Received Pre only	**3 ** ***(1.6)*** **°**	**4 ** ***(2.2)*** **°**	**2 ** ***(1.1)*** **°**	**3 ** ***(1.6)*** **°**
	Received Post only	**7 ** ***(3.8)*** **°**	**21 ** ***(11.5)*** §	**8 ** ***(4.4)*** **°**	**16 ** ***(8.7)*** *

Pre = pre-hospitalization; Post = post-hospitalization.

**°Group 1** (45%): POTENTIALLY NON-ADHERENT, includes likely under treated and misdiagnosed.

§ **Group 2** (31%): POTENTIALLY ADHERENT, likely new diagnoses occurring at the time of hospitalization.

* **Group 3** (24%): ADHERENT.

### Readmissions

A total of 11 children and adolescents (6%) had at least one other hospital admission during the 12 months following the index hospitalization. Of these, 9 were girls and 2 boys, and their median age was 11.0 (IQR 10–11). In all, 4/11 and 9/11 subjects were in therapy, respectively, before and after the incident hospitalization. 1/11 and 4/11 subjects received a spirometry claim or specialist visit referral, respectively, before and after the incident hospitalization.

## Discussion

In this retrospective observational study on Italian children hospitalized for asthma, the most notable finding is the low adherence to GINA guidelines, in terms of therapy and management, during the pre and the post hospitalization periods. Results may suggest that for half of hospitalized subjects, inaccurate diagnosis, under-treatment/scarce compliance with asthma guidelines by physicians, and/or scarce compliance to therapy by patients/their parents occurred. In all these cases, hospitalization would be a proxy indicator of preventable poor control of disease, rather than a proxy indicator of severity. It seems that only one-fourth of the hospitalized children have been managed following the guidelines. We previously described low adherence to guidelines, based on anti-asthmatic prescriptions [10], [11] and disease management [12] in an overall (regardless hospitalization) asthmatic population of children. A better adherence to asthma guidelines after a patient’s hospitalization was expected. The main change found was the adding of LTRA to ICS+LABA (Table 2) in 20% of children already in therapy before hospitalization. The analysis of the prescribed drug therapies introduced only after hospital admission revealed that one third of subjects who had no therapy before hospitalization received ICS+LABA as the first treatment. GINA guidelindes recommend adding LABA only when asthma is not controlled on low to high doses of ICS. In this study, the ICS+LABA claims received after four days, average hospitalization length, seem inappropriate because four days is a time not sufficient enough to test the efficacy of ICS monotherapy, before adding LABA. ICS+LABA as initial therapy was also reported in a US paediatric population [14]. Although no differences in serious adverse events were found between ICS+LABA and ICS alone [15], and in adults on low to high doses of ICS alone, the addition of a LABA reduces the rate of exacerbations and improve lung function ansymptoms [16], in steroid-naive patients with mild to moderate airway obstruction, the combination of ICS and LABA does not significantly reduce the risk of exacerbations requiring rescue oral corticosteroids comparing with a similar dose of ICS alone. For children no firm conclusions can be drawn regarding combination therapy in steroid-naive children, given the small number of children contributing data [17]. A low percentage (30%) of asthmatic children in the Lombardy Region undergoing spirometry during 1-year period was previously found [12], and the extent of spirometry utilization for disease monitoring and diagnosis was similar to other non-Italian paediatric populations [18], [19]. Since it was possible that spirometry testing was performed during the specialist visit, subjects receiving a spirometry claim and/or a specialist visit referral were calculated, and the percentage rose from 30 to 42%. In this study population, during the 12 months after hospitalization for asthma, a rate of lung function monitoring higher than 30% (42% considering also specialist visits) was expected. In fact, 38.5% of the hospitalized subjects received a spirometry claim after hospitalization and, considering the cases of spirometries performed during specialist visits, the percentage rose from 38.5 to 51%. However, the increase in spirometry testing found in the hospitalized population is not satisfactory yet, since 42% of the subjects never received a claim for spirometry or a specialist visit referral, before or after hospitalization, and this highlights a low compliance with guidelines in the monitoring of childhood asthma. The evaluation of appropriateness (Table 3) revealed that only 24% of subjects received adequate therapy and monitoring. By retrieving only incident hospitalizations, the study population would easily comprise, along with already diagnosed children, newly diagnosed subjects, likely diagnosed during hospitalization. The estimated rate of these cases was 31%, leaving a portion of hospitalized children/adolescents who are not appropriately treated and managed that is still nearly half. Although spirometry testing has not been correlated to minor hospitalization [18], the recent study TENOR (The Epidemiology and Natural History of Asthma: Outcomes and Treatment Regimens), reports the frequency of exacerbation outcomes in children aged 6 to 11 years and adolescents and adults aged 12 years and older stratified by lung function. A FEV1≤80% of the predicted value is associated with a double rate of hospitalization, versus a FEV1>80% of the predicted value [20].

The main limits of this study are the lack of information about the care received by the subjects while hospitalized, the missing diagnosis of asthma by the doctor, the absence of dosage details in the prescriptions, which made defining a real ‘step up’ modification of therapies difficult. Moreover, with this strategy it is possible to estimate the doctor’s lack of compliance to guidelines, but not the patient’s lack of compliance to therapy.

The findings of this study, i.e. that nearly half of children and adolescents in the 6–17 age range with an incident hospitalization for asthma did not receive any anti-asthma therapy during the 12 months before hospitalization, that 23% of them did not receive any therapy after hospitalization, and that only half of the subjects received a spirometry claim and/or specialit visit referral after hospitalization, are suggestive of an inaccurate diagnosis of asthma, a lack of compliance to guidelines by the primary physician, and/or a lack of compliance to therapy by asthmatic children/their parents. This latter may be due to lack of asthma education by doctors, to discontinuation of controller drugs as soon as symptoms resolve, and/or to concerns about side effects of long term controller therapy.

Findings are in agreement with a recently reported study [2] that about one fourth of pediatric hospitalizations that are potentially preventable with more adequate outpatient care is represented by asthma.

In conclusion, as for previous analyses of paediatric asthmatic populations, also in the hospitalized for asthma population described in this paper, adherence to asthma guidelines is low. The study described in this article was a pilot study and a longer observation period in a larger population would permit the estimation of preventable and non preventable (adequately cared for, but nonetheless hospitalized) cases of paediatric asthma.
